# The effect of gabapentin and pregabalin administration on memory in clinical and preclinical studies: a meta-analysis and systematic review

**DOI:** 10.1186/s12888-023-04696-x

**Published:** 2023-04-17

**Authors:** Zahra Behroozi, Maral Jafarpour, Maryam Razmgir, Sepideh Saffarpour, Hanieh Azizi, Ali Kheirandish, Tahereh Kosari-rad, Fatemeh Ramezni, Atousa Janzadeh

**Affiliations:** 1 Physiology Research Center, Institute of Neuropharmacology, Kerman University of Medical Sciences, Kerman, Iran; 2 The International Campus of Medicine, Iran University of Medical Sciences, Tehran, Iran; 3 Department of Medical Library and Information Sciences, School of Health Management and Information Sciences, Iran University of Medical Sciences, Tehran, Iran; 4 Department of Microbiology, Shahr-E-Qods Branch, Islamic Azad University, Tehran, Iran; 5 Medical University of Pécs Hungary, Pécs, Hungary; 6 BG Unfall Klinik, Frankfurt, Germany; 7 Department of Pharmacology, Faculty of Pharmacy, Mazandaran University of Medical Sciences, Sari, Iran; 8 Radiation Biology Research Center, Iran University of Medical Sciences, Tehran, Iran; 9 Physiology Research Center, Iran University of Medical Sciences, Tehran, Iran

**Keywords:** Gabapentin, Pregabalin, Memory, Pain

## Abstract

**Background:**

Today**,** gabapentinoids such as Gabapentin (GBP) and pregabalin (PGB) are widely used as painkillers. This may alter the function of the nervous system; hence their results may include a difference in memory and processes that end in memory formation. This study aims to conclude whether gabapentinoids can alter memory or not by reviewing and analyzing clinical and preclinical studies.

**Material and methods:**

A comprehensive search was carried out in databases including PUBMED, EMBASE, SCOPUS, and Web of Science. In the included studies, memory was measured as an outcome variable in clinical or preclinical studies.

**Result:**

A total of 21 articles (4 clinical, 17 preclinical) were included in the meta-analysis by STATA Software. The results showed that memory changes under the influence of GBP. Both the administrated dosage and the time of administration are important in the final results and latency time of retention. GBP administration in healthy animals increased latency time, whereas if the administration of GBP took place exactly before training, the latency time increased slightly.

Short-term administration of PGB in healthy volunteers is accompanied by transient side effects on the CNS. However, the number and homogeneity of the studies were not such that a meta-analysis could be performed on them.

**Conclusion:**

Clinical and preclinical studies showed that PGB administration did not confirm its improving memory effect. GBP administration in healthy animals increased latency time and improved memory. Although it depended on the time of administration.

## Introduction

Although all people experience pain, many patients suffered chronic pain, which is so excruciating and affects the patient's quality of life [1–3]. Various chemicals [4]and natural substances [5, 6] and new treatment methods such as photobiomodulation therapy [7, 8] have shown analgesic effects but their administration is not approved.

Gabapentin (GBP) and Pregabalin (PGB) are two drugs belonging to the gabapentinoid family. They are anticonvulsant drugs that have been used as an anti-nociceptive since 2004 [9, 10]. Now they are being used as a medication for diabetic neuropathy, neuralgia trigeminal, fibromyalgia, and even anxiety control [9]. GBP reduces monoamine neurotransmitters such as dopamine, and noradrenaline via changes in monoamine metabolism and/or in calcium channels related to releasing in pre-synaptic membranes [11, 12].

Voltage-gated calcium channels (VGCCs) that are made up of 3 subs units, α2δ, α, and b, were observed through CNS, and PNS are involved in calcium homeostasis, gene expression, and neurotransmitter release [13]. Although the Maximum density of VGCCs was discovered in the hippocampus and pyramidal and molecular cell layers of the cortex they were observed in the spinal cord and the dorsal root ganglions [10, 11, 14, 15]. VGCCs are involved in the regulation of sleep, circadian rhythm memory, and cognition [16]. Some processes involved in memory forming such as long-term potentiation (LTP) are dependent on the existence and function of the VGCCs) [17].

Gabapentinoids attach to α2δ subunits of the VGCCs in the presynaptic neurons of the hippocampus and layers of the cortex and down-regulated the expression of α2δ subunits, as the result, the pain is probably alleviated by reducing VGCCs activity [8].GBP detracts from neural excitation by increasing the release of gamma-aminobutyric acid (GABA) neurotransmitters [18, 19] [18, 20] which reinforces inhibitory control of GABA and modulates pain signaling [21]. Although GABA release is said to have a dampening effect on learning [22].

Another GBP pain-controlling mechanism is decreased amount of glutamic acid in the CNS [23]. GBP not only affects the metabolism of glutamate but also decreases the release of glutamate specifically in the posterior insula, a crucial place for maintaining memory [18, 23–26].

Therefore, since the mechanisms, sites of pain transmission, memory formation, and gabapentinoids function are similar in some aspects, it is speculated that GBP and PGB might affect memory formation through their performance.

Although studies have revealed the administration of some drugs involved in GABA mechanisms of function, can damage memory by harming the neural pathways responsible for controlling cognition [27–29], some preclinical studies of the effect of PGB on memory, have proven otherwise [22, 27, 30–35].

In this study, we aim to evaluate the effects of GBP and PGB on memory in both clinical and preclinical studies by meta-analysis. Because the usage of these drugs has been and still is increasing day by day. We hope our results be effective enough in the health care system and help patients suffering from pain.

## Material and methods

Five electronic databases were used to identify relevant studies including PubMed, SCOPUS, Web of Science, Embase, and Google Scholar. An example of the Pub Med search strategy is shown in Table 1. Searches included all studies up to December 20, 2022, and language restrictions were not applied. Given that, the same search strategy does not work in different databases, a separate search was written for each database.

**Table 1:** The designed search strategy of applied keywords in PubMed

(“Memory” [mesh] OR “spatial Memory” [mesh] OR “Memory, Episodic” [mesh] OR “Memory, Long-Term” [mesh] OR “Memory and Learning Tests” [mesh] OR “Memory Consolidation” [mesh] OR “Amnesia, Anterograde” [mesh] OR “Amnesia, Retrograde” [mesh] OR “Learning” [mesh] OR “Long-Term Memories” [tiab] OR “Long-Term Memory” [tiab] OR “Long term Memories” [tiab] OR “Long-term Memory” [tiab] OR “Long term Memory” [tiab] OR “Remote Memories” [tiab] OR “Remote Memory” [tiab] OR “Memory Disorder*” [tiab] OR “Memory and Learning Tests” [tiab] OR “Test of Memory and Learning” [tiab] OR “Memory Consolidation” [tiab] OR “Metacognition” [tiab] OR “Meta-cognition” [tiab] OR “Meta cognition” [tiab] OR “Metacognitive Awareness” [tiab] OR “Meta-cognitive Awareness” [tiab] OR “Meta cognitive Awareness” [tiab] OR “Meta-cognitive Monitoring” [tiab] OR “Metacognitive Control” [tiab] OR “Metaemotion*” [tiab] OR “Meta-emotion” [tiab] OR “Meta emotion”[tiab] OR “MetaMemor*” [tiab] OR “Meta-Memory” [tiab] OR “Meta Memory” [tiab] OR “Meta-memories” [tiab] OR “Amnesia” [tiab] OR “Anterograde Amnesia*” [tiab] OR “Anterograde Memory Loss” [tiab] OR “Retrograde Amnesia” [tiab] OR “Retrograde Memory Loss” [tiab] OR “Repression” [tiab] OR “Repressed Memory” [tiab] OR “Delayed Memory” [tiab] OR “Delayed Memories” [tiab] OR “Amnesia Memory Loss” [tiab] OR “Amnestic State” [tiab] OR “Tactile Amnesia*” [tiab] OR “Temporary Amnesia*” [tiab] OR “Dissociative Amnesia” [tiab] OR “Dissociative Amnesia” [tiab] OR “Photographic Memory” [tiab]) AND (“Gabapentin” [mesh] OR “Gabapentin” [tiab] OR “Neurontin” [tiab] OR “Convalis” [tiab] OR “Pregabalin” [mesh] OR “Pregabalin” [tiab] OR “Lyrica” [tiab] OR “Gabapentinoid” [tiab] OR “Neurontin” [tiab] OR “Horizant” [tiab] OR “Gralise” [tiab] OR “3 isobutyl GABA” [tiab] OR “CI1008” [tiab])	

### Exclusion criteria

The following studies were excluded from our study: Review articles, in-vitro studies, and articles not sufficiently relevant to experimental memory. For example, they had measured molecular factors related to memory such as BDNF, but they had not investigated memory with behavior, and, they did not report the control group, error bar, or any other statistical tribulation that distorted the results.

### Inclusion criteria

The following studies were used in our study: Preclinical peer-reviewed studies based on gabapentin and pregabalin administration and memory evaluation, peer-reviewed studies based on the patient using gabapentin or pregabalin and had memory test results, and Studies that used a healthy or control group in addition to the patient group, Short-term, and long-term memory were measured by validated tests.

## Methods of study assessment

Initially, two co-authors independently screened the studies that were returned by the searches based on title and abstract. Where there was doubt, the full text of the article was inspected. Conflicting eligibility determinations were decided by consensus. A third reviewer was invited to resolve disagreements between the 2 reviewers [36–38].

### Risk of bias assessment

Quality assessment for clinical studies is performed by the Higgins method [39] (Table 2). The risk of Bias (ROB) tool adapted for animal studies was also used to objectively assess the quality of the preclinical studies that met the inclusion criteria [29] (Table 3). This scale consists of 10 items assessing 5 broad categories (Table 3). The following scale was used to convert the quantitative measure obtained into a qualitative assessment: < 50% (weak), 50%–69% (fair), 70%–79% (good), and 80%–100% (very good). Two independent assessors completed the form for each study and their answers were compared. Any disagreements were resolved through discussion or by involving a third reviewer.

**Table 2:** Quality evaluation of the clinical trial (Higgins method)

Selection bias: ✓ = biased allocation to comparison groups
Performance bias: ✓ = unequal provision of care apart from treatment under evaluation
Detection bias: ✓ = biased assessment of outcome
Attrition bias: ✓ = biased occurrence and handling of deviations from protocol and loss to follow up
External validity—the extent to which results of trials provide a correct basis for generalization to other circumstances ✓ Patients: age, sex, the severity of disease and risk factors, comorbidity ✓ Treatment regimens: dosage, timing, and route of administration, type of treatment within a class of treatments, concomitant treatments ✓ Settings: level of care (primary to tertiary) and experience and specialization of care provider ✓ Modalities of outcomes: type or definition of outcomes and duration of follow up

**Table 3:** Characteristics evaluated preclinical studies based on guidelines of the agency for healthcare research and quality methods guide for effectiveness and comparative effectiveness reviews

1) Species,2) Strain,3) Age/Weight, 4) Genetic Background, 5) Number of animals per group, 6) Definition of Control, 7) Method of Allocation to Treatments, 8) Target Tissue Using,9) Appropriate Tests, 10) Blindness of Assessor,11) Randomization,12) Definition of the experimental unit (individual animal/animals in one cage), 13) Description of Statistical, 14) animal facility,15) Ethics,16) Description of the Reasons to Exclude Animals from the Experiment during the Study	

### Analysis

The primary aim of this study was to investigate memory under the influence of gabapentinoids(GBP and PGB) administration**.** Methods of assessing memory in clinical studies were different: Psychomotor Vigilance Task (PVT) [40], Rey-Auditory Verbal Learning Test (RAVLT) [40], The Brief Visuospatial Memory Test-Revised (BVMT-R) [40], Wechsler Memory Scale-Revised [29], The Rivermead Behavioral Memory Test [29], Benton Visual Retention Test [29], Instruction A, vigilance (Rapid Visual Information Processing, RVIP) [41] and, serial memory scanning (Sternberg Short-Term Memory Scanning Test, STM) [41]. Only one study examined the effect of GBP on memory in orthopedic surgery patients and for this purpose, using a picture recall test of Snodgrass and Vander wart. To evaluate memory in the preclinical study, five studies used the Morris Water Maze test [39, 42–45] and ten studies used the passive avoidance test [28, 46–53]. One study used delayed a matching-to-sample (DMTS) task [54]. Each Y-maze and social recognition memory test was used in one study [55]. Three studies used object recognition memory [56–58]. One study used an Open field test [42]. One study used spontaneous alternation behavior to assess memory changes [59]. Data obtained from the analysis of memory tests the data were presented as mean and standard deviation (SD). STATA 14 was used for data analysis. Effect size with a 95% confidence interval (95% CI) was calculated. The fixed-effect model was applied and if the heterogeneity was ≥ 50%, the random-effect model was used.

## Result

**Fig. 1:**
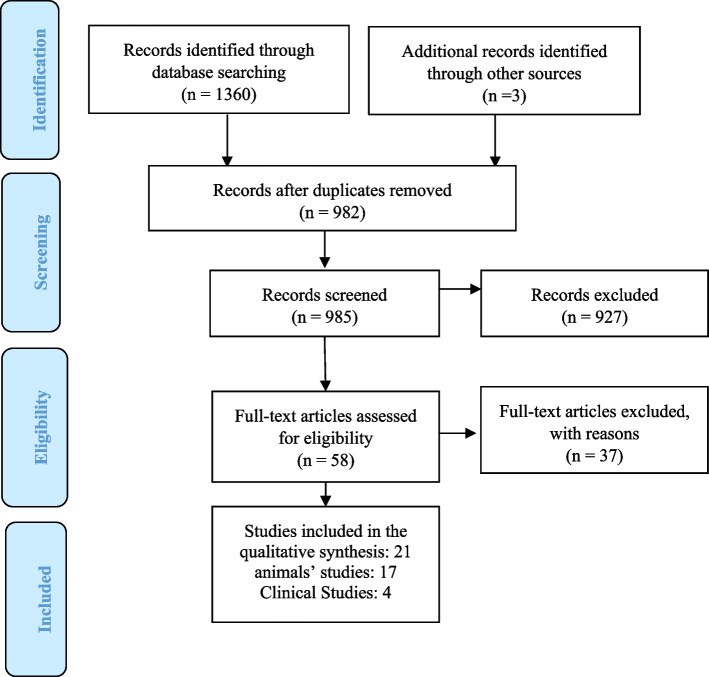
Prisma flow chart shows the process of identification and selection of studies about Gabapentin and Pregabalin on memory

This search returned 1360 results from 123 from PubMed, 710 from EMBASE,383 from SCOPUS, and 144 from Web of science were selected for more investigations. After removing the duplicates 982 articles remained (Fig. 1).

The final evaluation of preclinical studies revealed 21 animal studies, 17 studies for GBP, and 4 studies about the effect of PGB on memory. Two studies examined the effect of GBP on memory in normal animals [50, 51]. One study was performed on the anxiety model [28]. One study used animals exposed to tobacco smoke during fetal life [39]. Five studies selected the peripheral neuropathy model (CCI, SNL, diabetic) [44, 54, 59, 60]. Eight studies were performed their study in the epilepsy model [42, 43, 47–49, 51, 53, 58]. For seizure induction, different models were selected including one study of Kainic acid [42] and four study PTZ [43, 47, 51, 58]. Among these studies, two studies used two methods to induce seizures. One of them used PTZ and the Increasing current electroshock (ICES) method [58]. Another study used LiCl and pilocarpine [43] (Table 4).

**Table 4:** Abstract of all preclinical articles evaluated the effect of Gabapentin or Pregabalin administration on memory

Author Year	animal /gender/Weight(g)	Animal model	Time of Study	Learning Memory test	Administration method	Dosage
Gabapentin						
Acosta/2000 [53]	CF-1 mice /male/ 25 ± 30	seizure	1 day	Inhibitory avoidance	IP/30 min before training, immediately after training 180 min after training with food shocked	5, 10, 50, and 100 mg/kg
Blake/2004 [28]	CF-1 mice/male /25 ± 30	seizure	1 day	Inhibitory avoidance	IP/ immediately after training/ 20 min after training	10 and 100 mg/kg
Blake/2007 [52]	CF-1 mice/ male /25 ± 30	anxiety	14 days	Inhibitory avoidance	IP/ immediately after training/ Twice a day for 7 days after training	50 mg/kg
Blake/2010 [51]	CF-1 mice/ male /25 ± 30	Epilepsy (PTZ)	24 days	Inhibitory avoidance	Implantable GBP-loaded DDS/ Release for 7 days	4.5 mg/day
Boccia/2001 [61]	CF-1 mice/ male/ 25 ± 30	Normal	1 day	inhibitory avoidance	IP/10 min after training/ Single-dose	10 and 5 mg/kg
Buccafusco /2010 [54]	PG/female and male/1.82 kg	Diabetic NP	1 day	(DMTS) task/ working memory	IM/ 30 min before testing	1, 3, 10, and 30 mg/kg
Cilio /2001 [42]	SD rats /male/ neonatal	Epilepsy	16 days	Water maze, Open field test	IP/ 24 h after onset of SE	200 mg/kg/ twice daily/ 100 mg/kg/ twice daily 50 mg/kg
Czubak /2008 [39]	Wistar rats/ Female / 180–200	exposure to tobacco smoke (fetal life)	21 days	Morris Water Maze Test	IP/ Single administration GBP for 7, 14, and 21 days	12.5, 25, and 50 mg/kg
de-Parisa /2000 [50]	Wistar rats/ male 250–350	Normal animals	1 day	Step-down inhibitory avoidance task	IP / 30 min before each behavioral task	10,30,100 mg/kg
Goel /2011 [58]	Albino Swiss mice/male/ 18–30	Epilepsy	4 days	Object recognition test	Oral/ 4 days	50 and 100 mg/kg
Grégoire /2012 [55]	SD rats /mail/50–175	NP (CCI)	21 days	Y-maze Social recognition memory test	Oral/ 60 min before the behavioral test	3–10–30 mg/kg
Gulec Suyen /2016 [43]	Wistar rat/ male /300–350	Status Epilepsy	44 days	Morris water maze	ICV /7 days treatment 6 month delay after epilepsy + 7 days treatment	100 μg/10 μl
Jayarajan/ 2015 [59]	Wistar rats/ male/ 7–10 weeks	NP (SNL)	21 days	Contextual fear condition	IP/ 14–21 days after the surgery	30 and 300 mg/kg
Krawczyk /201 [49]	Swiss mice /male/22–27 g	Seizure by MES	16 days	step-through passive avoidance task	IP/ 60 min before tests	200 mg/kg
Khaled G. Abdel-Wahhab /2018 [44]	albino rats/ 150–200 g	diabetic NP	20 weeks/ high-fat diet for 12 weeks + 8 weeks of treatment	Morris Water Maze Test	Oral/ daily for 8 weeks	20 mg/kg
Jastrzebska /2009 [48]	Swiss mice/male/ 22–26 g	Seizure	NO	Step-through passive avoidance task	IP/ 240 min before seizures and behavioral tests as well as before brain sampling	0.005 ml/g body weight
Borowicz /2002 [47]	Swiss mice /male/ 20–25 g	Epilepsy	1 day	Passive avoidance task,	IP/ 60 min before electro convulsions and behavioral tests	25/50/100/200 mg/kg
Pregabalin						
Chen /2017 [45]	SD rat/male/200–260	Chronic trigeminal neuralgia	53 days	Morris water maze test	Intra-gastric administration	30 mg/kg
Kawano /2016 [57]	Wistar rat /male/ 585–650	Abdominal surgery	days14	The novel object recognition task	IP/ Early treatment:1 h before surgery 3- or 7-days’ Late treatment. (4–13 days)	10 mg/kg
La Porta /2016 [56]	Swiss albino mice /male/8–12 weeks	NP( SNL)	27 days	Object recognition memory	IP/ Early treatment 30 min before surgery. (day 27) twice daily	20 mg/kg
Routt /2018 [46]	Inbred Swiss mice/ female / 20–25 g	Epilepsy by MES	1 day	Passive-avoidance task	IP / 120 min before all tests	161.4 and 104.2 mg/kg

*NO* not reported, *IP* Intraperitoneal, *I.C.V* intra-cerebro ventricular injection, *C.C.I* chronic constriction injury, *NP* Neuropathic Pain, *DMTS* Delayed Matching-to-Sample, *MES* Maximal electroshock, *PG* pigtail macaques, *SD* Sprague Dawley

Four clinical studies [29, 40, 41, 62] were found, one study was about GBP [62], and, three studies were about PGB administration [29, 40, 41]. The subjects selected in the human study for evaluation of the PGB effect on memory status were different and included: partial epilepsy and insomnia [40], refractory partial epilepsy [29], and Healthy volunteers [41] (Table 5).

**Table 5:** Abstract of clinical studies evaluating the effect of Gabapentin or Pregabalin on memory

Author Year	Gender/Weight/ Age	Spices/ number	Pain disease	Administration method	Dosage	Duration of study	Memory test	Conclusion
Gabapentin								
F. Adam /2012 [62]	male and female/BMI lower than 30 kg/m2/16–74 year	American /64	orthopedic surgery / open inguinal hernia repair	Oral/ 3 h before the anesthesia	1200 mg	NO	Preoperative memory performance/ postoperative memory performance	Oral premedication with 120GBP reduced anxiety
Pregabalin								
Ian Hindmarch /2005 [41]	male and female/ 24.4 kg/m2 BMI/ 29 years	96% white Caucasian	Healthy volunteers	Oral/ for 3 days	150 mg (T.I.D)	87 days	vigilance (RVIP)/serial memory scanning (STM)	PGB did not differ on most assessments from placebo
Anne-Sophie Ciesielski /2006 [29]	male and female /No/22–52 years	NO	refractory partial epilepsy	Oral/ 14 days	300 mg	14 days	Wechsler Memory Scale-Revised/River-mead Behavioral Memory Test/Benton Visual Retention Test	Higher anxiety scores and higher variability in hostility scores with PGB than levetiracetam
C.W. Bazil /2012 [40]	male and female/ no/20–49 years	NO	Partial epilepsy and insomnia	Oral/ 14 days	150 mg BID	28 days	Psychomotor Vigilance Task (PVT).Rey-Auditory Verbal Learning Test (RAVLT)/The Brief Visuospatial Memory Test-Revised (VMT-R)	Pregabalin administration results in no reduction of memory. The low number of participants in this the study could be the reason for this finding

*NO* not reported, *T.i.d* three times a day, *RVIP* Rapid Visual Information Processing, *STM* Sternberg Short-Term Memory Scanning Test

The quality control results of preclinical studies are shown in Table 6(GBP and PGB). In the qualitative review of pre-clinical studies, it was found that the lowest quality was 56.25% [58] [48] (fair quality)and the highest quality was 93.75(very good quality) [57]. Most of the studies received a negative score from the description of the reasons to exclude animals from the experiment during the study, appropriate tests, and the blindness of the assessor. Basic characteristics of animals such as Species, Strain, Age/Weight, Genetic Background, number of animals per group, and definition of Control, were mentioned in most of the studies (Tab 6).

**Table 6:** Quality assessment of preclinical articles for gabapentin and pregabalin

Gabapentin																	
Author Name/YEAR	1	2,	3	4	5	6	7	8	9	10	11	12	13	14	15	16	./
G. Acosta/2000 [53]	Y	Y	Y	Y	Y	Y	Y	N	N	Y	N	N	Y	Y	N	N	68.75
M. Blake/2004 [28]	Y	Y	Y	Y	Y	Y	Y	N	Y	Y	N	Y	Y	Y	Y	N	81.25
M. Blake/2007 [52]	Y	Y	Y	Y	Y	Y	Y	Y	Y	Y	N	Y	Y	Y	N	N	81.25
M. Boccia/2001 [61]	Y	Y	Y	Y	Y	Y	Y	N	Y	Y	N	N	N	Y	N	N	62.5
K Borowicz / 2002 [47]	Y	Y	Y	Y	Y	Y	Y	Y	Y	N	Y	Y	Y	Y	Y	N	87.5
J. Buccafusco/ 2010 [54]	Y	Y	Y	Y	Y	N	Y	N	Y	N	N	Y	Y	Y	Y	N	68.75
M. Cilio/ 2001 [42]	Y	Y	Y	Y	Y	Y	Y	N	Y	N	Y	Y	Y	Y	Y	Y	87.5
A. Czubak/ 2008 [39]	Y	Y	Y	Y	Y	Y	Y	N	Y	Y	Y	Y	Y	Y	Y	N	87.5
F. de-Parisa/ 2000 [50]	Y	Y	Y	Y	Y	Y	N	N	N	N	N	Y	Y	Y	N	N	56.25
M.-Jastrzebska /2009 [48]	Y	Y	Y	Y	Y	Y	Y	Y	N	N	Y	Y	Y	Y	Y	Y	87.5
R.Goel/ 2011 [58]	Y	Y	Y	Y	Y	Y	N	Y	N	N	N	N	Y	Y	Y	N	56.25
S.Grégoire/ 2012 [60]	Y	Y	Y	Y	Y	Y	N	Y	Y	Y	N	Y	Y	Y	Y	N	81.25
G. Gulec Suyen /2016 [43]	Y	Y	Y	Y	Y	Y	Y	N	Y	N	Y	Y	Y	Y	Y	Y	87.5
M -Krawczy/ 2016 [49]	Y	Y	Y	Y	Y	Y	Y	Y	N	N	Y	N	Y	Y	Y	Y	81.25
Pradeep Jayarajan/ 2015 [59]	Y	Y	Y	Y	Y	Y	Y	Y	N	Y	N	Y	Y	N	Y	N	75
**Pregabalin**																	
C. La Porta/ 2016 [56]	Y	Y	Y	Y	Y	Y	Y	N	Y	Y	Y	Y	Y	Y	Y	N	87.5
T. Kawano/ 2016 [57]	Y	Y	Y	Y	Y	Y	Y	Y	Y	Y	Y	Y	Y	Y	Y	N	93.75
K.Reutt/ 2018 [46]	y	Y	Y	Y	Y	Y	Y	Y	Y	N	Y	N	Y	Y	Y	N	81.25
R.Wen Chen/ 2017 [45]	Y	Y	Y	Y	N	Y	N	N	Y	Y	Y	Y	Y	Y	Y	Y	81.25

1) Species,2) Strain,3) Age/Weight, 4) Genetic Background, 5) Number of animals per group, 6) Definition of Control, 7) Method of Allocation to Treatments, 8) Target Tissue Using,9) Appropriate Tests, 10) Blindness of Assessor,11) Randomization,12) Definition of the experimental unit (individual animal/animals in one cage), 13) Description of Statistical, 14) animal facility,15) Ethics,16) Description of the Reasons to Exclude Animals from the Experiment during the Study

### The result of quality control for clinical studies are in Table 7 for GBP and PGB

**Table 7:** Quality assessment of clinical articles (score based on Higgins’s method), Gabapentin, and Pregabalin

Gabapentin																		
Study	1	2	3	4	5	6	7	8	9	10	11	12	13	14	15	16	17	%
F. Adam/ 2012 [62]	✓	×	✓	✓	✓	✓	✓	✓	✓	✓	✓	×	×	✓	✓	✓	✓	82.3%
Pregabalin																		
Study	1	2	3	4	5	6	7	8	9	10	11	12	13	14	15	16	17	%
C.W. Bazil/ 2012 [40]	✓	×	✓	✓	✓	✓	✓	×	✓	✓	✓	×	×	✓	✓	✓	✓	76.4%
Anne-Sophie Ciesielski/ 2006 [29]	✓	×	✓	✓	×	×	✓	×	✓	✓	✓	✓	×	✓	✓	✓	✓	70.5%
Ian Hindmarch/ 2005 [41]	✓	✓	✓	✓	✓	×	✓	×	✓	✓	✓	×	×	✓	✓	✓	✓	76.4%

1-gender, 2-spices, 3- duration of follow-up, 4- statistic description, 5-Blinding care, 6-Blinding of patients, 7-Clear inclusion/ exclusion criteria, 8- Route of administration, 9- timing, 10- type or definition of outcomes, 11-Randomization, 12-Appropriate tests, 13-Raw data available, 14-dosage, 15-Methodological, 16- quality comparable group, 17-Negative positive controls

In the qualitative review of clinical studies, the score of the articles was between 70.5 and 823./. (Good quality). Most of the articles about the route of administration, raw data availability, spices, and appropriate tests did not mention and got a negative score (Tab 7).

**Fig. 2:**
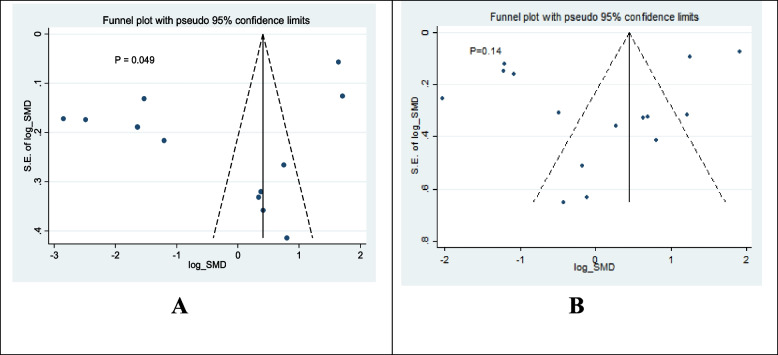
Funnel plot to asymmetry test for the evaluation of the publication bias in studies that examined (A) latency time on retention, and (B) seizure in animals receiving GBP compared with untreated animals

In the present study, small-study bias was observed in the studies that investigated the effect of GBP on the Latency time (*p* = 0.049) (Fig. 2A). Any small-study effect was not found in the studies that investigated the effect of GBP on seizure (Fig. 2B) (*p* = 0.14).

### Meta-analysis

**Fig. 3:**
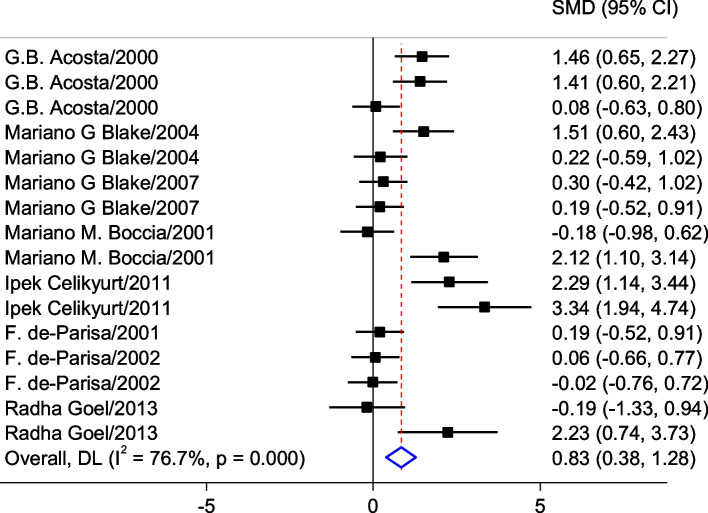
Forest plot of screening characteristics of the effect of gabapentin (GBP) administration on latency time on retention in Preclinical studies

In a general analysis, the data of latency time to retention (in the Passive avoidance test) with different administrated dosages and different used curing protocols have been evaluated. The result showed GBP has a medium effect on the memory of a normal animal since the latency time to retention increased after GBP administration (SMD = 0.83; 95% CI: 0.38 to 1.28; *p* < 0.0001) which means the memory has improved moderately(Fig. 3). Afterward, the analysis was done based on the different administrated dosages and curing protocols.

An analysis of a subgroup in which a drug less than 10 mg/kg was used (SMD = 1.163; 95% CI: 0.44 to 1.88; *p* = 0.0002) revealed that the latency time is more than the subgroup which received 30–100 mg/kg of the drug (SMD = 0.5; 95% CI: -0.02 to 1.02; *p* = 0.06). In analysis subgroups based on pre or post-training administration, in the pre-training subgroup (SMD = 1.10; 95% CI: 0.19 to 2.01; *p* = 0.018) the latency time is more than the post-training subgroup (SMD = 0.69; 95% CI: 0.18 to 1.20; *p* = 0.016) (Table 8).

**Table 8:** Subgroup analysis of Gabapentin administration effect on the memory of treated animals compared to non-treated animals

Subgroup	Number of experiments	Heterogeneity (*p*-value)	SMD (95% CI)	*P* -Value
Dose (mg/kg)				
< 10	8	81.6% (< 0.0001)	1.163 (0.44 to 1.88)	0.002
> 10	8	65.8% % (0.005)	0.5 (-0.02 to 1.02)	0.064
Pre or post-training treatment				
Pre	6	84.8% (< 0.0001)	1.10 (0.19 to 2.01)	0.018
Post	10	71.1% (< 0.0001)	0.69 (0.18 to 1.20)	0.016

*CI* Confidence interval, *SMD* Standardized mean the difference

Foot shock induction time was also considered as a criterion for subgroup analysis (Table 9). Studies were divided according to whether training was performed before, after, or immediately after foot shock. The results showed that training immediately after the shock has a moderate (SMD = 0.61; 95% CI: 0.02 to 0.72; p = 1.19) effect on latency time.

**Table 9:** Subgroup analysis of Foot shock induction time, before or after training on latency time in Gabapentin-treated animals

**Pre or post-Foot Shock induction**	Number of experiments	Heterogeneity (*p*-value)	SMD (95% CI)	*p*-value
Pre	7	85.9% (< 0.0001)	-0.69 (-1.86 to 0.49)	0.252
immediately	6	64.1% (0.016)	0.61 (0.02 to 1.19)	0.041
Post	3	82.7% (0.003)	0.64 (-0.69 to 1.97)	0.343

*CI* Confidence interval, *SMD* Standardized mean difference

### The effect of gabapentin on memory in epilepsy situation

**Fig. 4:**
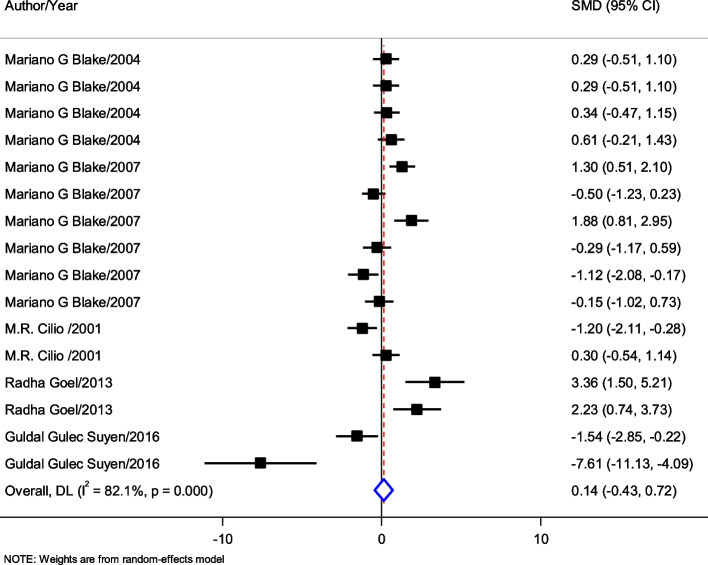
Forest plot of screening characteristics of the effect of gabapentin (GBP) administration on memory in the epileptic situation in preclinical studies

In 5 studies and 16 experiments, the effects of GBP on the memory of epileptic animals have been studied. GBP doesn’t show a significant impact on memory in epileptic animals (SMD = 0.14; 95% CI:—0.43 to 0.72; *p* = 0.27) (Fig. 4).

The analysis of subgroups is based on the administrated dosage (Table 10). The subgroups include less than 10 mg/kg, 50 mg/kg, and more than 100 mg/kg. Also, the analysis of the outcomes based on the time of drug administration revealed that administration of GBP before, immediately after, and after the training, doesn’t make a significant difference in latency time.

**Table 10:** Subgroup analysis of GABA treatment effect on memory function of epileptic animals compared to normal animals

Subgroup	Number of experiments	Heterogeneity (*p*-value)	SMD (95% CI)	*P*-value
Dose (mg/kg)				
< 10	2	0.0% (< 0.0001)	0.38 (-0.25 to -0.9	0.276
50	7	85.3% (0.005)	0.50 (-0.43 to -1.43)	0.291
> 100	7	85.5% (0.005)	-0.39 (-1.48 to -0.70)	0.483
Pre or post-GBP treatment on training				
pre	6	90.2% (< 0.0001)	-0.35)-2.09 to 1.40)	0.696
immediately	4	0.0% (0.939)	0.39)-0.02 to 0.79)	0.063
post	6	83.1% (< 0.0001)	0.17 -)0.69 to 1.04)	0.696

## Discussion

As mentioned, many of the prescribed painkillers are indeed antiepileptic drugs such as GBP and PGB. Whilst designing the current meta-analysis and systematic review, we assumed that finding both preclinical and clinical studies would be easy and that there was enough research on it because these were drugs that induced crucial effects on brain function and have extensive usage. But it seems that this issue was ignored by researchers. However, we were left hopeless since the outcome of the research wasn’t convincing. The effect of these two drugs on the nervous system specifically on memory hasn’t been studied enough. We were excited to report the outcomes of clinical PGB and GBP studies on memory but there weren't even 3 of the same studies that checked the effect of these two drugs on chronic pain.

In this study, we primarily reported brief data concerning the effects of PGB and GBP separately in normal animals. Meta-analysis about GBP usage revealed that administration of this drug can increase the latency time in normal animals (based on the protocol it means, the improvement in memory function). However, it should be noted that our results are about rodents, and clinical studies are needed in this area.whereas, there was not much information available on the PGB effect on memory in normal and healthy animals so we couldn't present the results of the study by meta-analysis.

Drugs that reduce or blocked GABA-A receptors improved memory in rodents [63]. Today we know that the GABAA receptor has provided an excellent target for the development of drugs with an anticonvulsant action. GBP, a cyclic analog of GABA, acts by enhancing GABA synthesis and also by decreasing neuronal calcium influx via a specific subunit of VGCCs [56]. Memory processing requires tightly controlled signaling cascades, many of which are dependent upon intracellular calcium balance [64, 65].

In subgroups based on administrated dosage, latency time and memory increased in the animal group which received less than 10 mg/kg of the drug (SMD = 1.22). On the other hand, in the other subgroup of animals that received more than 10 mg/kg (30,50, and 100 mg/kg) of the drugs, no significant difference was revealed in the latency time. These results demonstrate the fact that the best possible outcome on memory can be reached in a lower dosage of the drugs and its higher dosage could be accompanied by adverse effects. Of course, this finding is in the condition that the method of drug administration was different in the studies, which is explained in Table 4 in the Administration method section. Because in some cases, seizures were induced or training was earlier than drug administration, and in others, this order was the opposite.

The time of training either before or after the seizure has an impact on memory, in a way that if the animal gets trained right after GBP administration, latency time increases slightly as a result of memory improvement. Despite this Outcome, GBP should be used with more attention to maintain and improve memory [39, 53]. Likewise, training time before or after seizure and foot shock in passive avoidance tests has an impact on memory in animals. If the training takes place right after or during epilepsy or foot shock induction, latency time and memory increase. However, if the animal gets trained right before seizure induction, no changes will be seen in memory. This may be because seizure, impairs memory by harming neurons [66], whereas taking antiepileptic drugs possibly either neutralize this effect or prohibit further damage [67, 68]. This study and the written search strategy are for the effects of GBP and PGB on memory and are not specified on the effect of seizures on memory. Therefore, we suggest more investigations in this regard.

In this systematic review and meta-analysis, we encountered studies with the opposite ideas. Despite this hypothesis, spatial learning triggers lasting increases in GABA release. Because GBP enhances the expression of δGABAA receptors [69].and Cui et al. [22]presented, that the use of released GABA impairs memory due to its effect on long-term potentiation, and learning [22]. Although the meta-analysis results did not support this theory, they suggest the need for more studies and stronger reasoning in this area.

Clinical results determined that GBP in non-epileptic patients has no significant impact on getting worse memory. One clinical study was performed on the effect of GBP administration on memory. Adam et al. examined the effect of 2 or 3 h, preoperative administration on anxiety, amnesia, and sedation with a limited number of patients and only in a short time [62]. GBP premedication provided a reduction in preoperative anxiety without causing sedation or impairing preoperative memory [62]. But long-time administration effect was not evaluated.

PGB is a potent ligand for the alpha-2-delta subunit of VGCCs in the central nervous system that prescribes as an anticonvulsant, Pain reliever, and anxiolytic agent [70, 71]. However, the published outcome on the effect of PGB administration on memory was contradictory.

Pain and chronic pain are a company by several cognitive disruptions, leading to problems in attention, spatial memory, recognition memory, and decision-making [72, 73]. Porta et al. reported the presence of neuropathic pain mice was associated with increased anxiety- and depressive-like behaviors, and reduced memory functions, chronic PGB treatment improved the nociceptive, anxiety-like, as well as memory deficit, but did not modify the depressive-like behavior [56]. While Chen and Kawano et al. indicated that PGB could not inhibit the cognition deficit formed by chronic pain [45, 57]. However, it stated, that if PGB is administered before surgery, could prevent cognitive dysfunction developed, after animal abdominal surgery. But it cannot cure cognitive impairment due to surgery pain [57].

Three-day administration of PGB in the lithium-pilocarpine model in rats indicated PGB did not differ on most assessments from placebo, producing only minor, transient impairment on some objective cognitive and psychomotor measures [53].

Clinical studies about the effects of PGB on cognition and attention reported improvements in sleep quality in epileptic patients, which coincided with improved attention, and a decrease in reaction time, although, fewer effects of interference on memory were published [29, 40, 44]. In this regard, the increase in slow-wave sleep was not sufficient to result in memory improvement [40].

## Conclusion

GBP administration in healthy animals increased latency time and improved memory, in this regard time of administration is important, If the administration of GBP takes place right before training, or during epilepsy or foot shock induction, latency time and memory increase slightly. However, if the animal gets trained right before seizure induction, GBP administration causes no changes in memory. GBP administration in patients with epilepsy had not reported adverse effects on memory, but studies were not sufficient to draw general conclusions about the effect of GBP on memory. Clinical and preclinical studies on cognition and memory following PGB administration did not confirm its positive memory effect. Reported short-term administration of PGB in healthy volunteers is accompanied by transient side effects on the CNS.

However, the number and homogeneity of the studies were not such that a meta-analysis could be performed on them. Our results demonstrated the importance of continuing to study anti-epileptic drugs and their side effects.

## Data Availability

All relevant raw data will be freely available to any scientist wishing to use them for non-commercial purposes, upon a reasonable written request from the corresponding author (AJ).
